# Phenols and Melanoidins as Natural Antioxidants in Beer. Structure, Reactivity and Antioxidant Activity

**DOI:** 10.3390/biom10030400

**Published:** 2020-03-04

**Authors:** Alvaro Martinez-Gomez, Isabel Caballero, Carlos A. Blanco

**Affiliations:** Dpto. Ingeniería Agrícola y Forestal (Area de Tecnología de los Alimentos), E.T.S. Ingenierías Agrarias, Universidad de Valladolid, 34004 Palencia, Spain; alvaromartinezgomez1@gmail.com (A.M.-G.); isabel.caballero@uva.es (I.C.)

**Keywords:** beer, phenols, melanoidins, antioxidant activity

## Abstract

Beer is one of the most consumed drinks around the world, containing a variety of compounds that offer both appreciated sensorial characteristics and health advantages. Important healthy compounds in beer are those with antioxidant properties that attenuate the content of free radicals produced as by-products in the human metabolism, exerting an appreciable effect against cancers or cardiovascular diseases. This work details a study of antioxidant compounds present in beer, focusing on the two main groups: phenols (including polyphenolic forms) and melanoidins, formed specifically during brewing as Maillard products. The fundaments of the most important methods to evaluate beer antioxidant activity, the main antioxidant compounds present in beer—especially those with healthy properties—and the new trends to increase beer antioxidant activity are also discussed.

## 1. Introduction

Beer, containing hundreds of different compounds, is one of the oldest, most consumed and popular drinks in the world. Some compounds are directly derived from raw materials and others are produced during brewing. Raw material sources of chemicals in beer are water, malt (along with adjuncts such as wheat, rice, corn or sugar), hops and yeast.

Beers are primarily classified according to the fermentation process [1]. Lagers, the most consumed type of beer, are produced by low fermentation, which is usually carried out between 6 and 15 °C. In contrast, ale-type beers are produced by high fermentation, occurring between 16 and 24 °C after which yeast cells rise to the surface of the fermentation media, forming a thick film that is not generally removed completely. Finally, lambic beer is the result of spontaneous fermentation.

In recent years, the nutritional interest of beer has increased because it is rich in antioxidant compounds with low ethanol content. Antioxidants are important compounds that help us stay healthy, attenuating the oxidative stress which arises from overproduction of reactive oxygen or nitrogen species (ROS/RNS). The collective terms “reactive oxygen species (ROS) and reactive nitrogen species (RNS)” have been applied to a variety of free radicals such as superoxide, hydroxyl, peroxyl, nitric oxide, nitrogen dioxide radicals, as well as to non-radical reactive intermediates like hydrogen peroxide (H_2_O_2_) and peroxynitrite (ONOO^−^). These free radicals are currently produced under normal physiological conditions in our organism, but its generation is exacerbated under pathological conditions, and they play an important role in pathological processes and regulatory activities. Antioxidants can act in different ways, they can scavenge free radicals, inhibit prooxidative enzymes, and chelate metal ions, among others [2].

**Figure 1:**
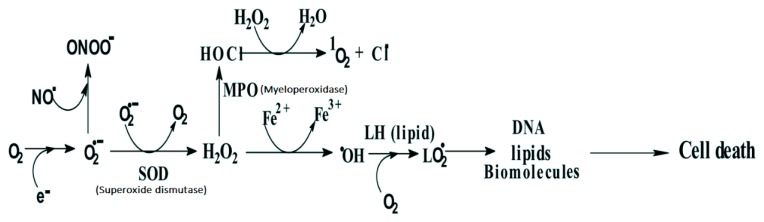
Chemical changes in a cell and reactive oxygen species (ROS) and reactive nitrogen species (RNS) involved. Adapted from [4].

Free radicals are fundamental to any biochemical process and represent an essential part of aerobic life of our metabolism. While superoxide radical is not so reactive and may not be able to cause any direct damage to cells, its reaction product, hydrogen peroxide, in the presence of trace metal ions such as Fe+2, is converted to hydroxyl radicals (•OH), which in turn can oxidize most of the biomolecules [3]. Organic substrates (RH) or lipids (LH), after their reaction with hydroxyl radicals in presence of oxygen are converted into peroxyl radicals (ROO^•^)/(LOO^•^), which are known to undergo chain reactions, and thereby multiply the damage. Thus, free radicals formed within the cells can induce multiple chemical changes in cellular organelles like lipid membrane, DNA and proteins, which can eventually lead to cell death (Figure 1).

Beer antioxidants mainly come from two ingredients used in brewing: malt and hop. The antioxidant capacity of beer therefore depends on the antioxidant contents in these two ingredients and on different parameters involved in brewing, namely the variety of barley, the malting process, temperature and pH during mashing, sparging, boiling, the variety of hops used and yeast fermentation.

Around 70–80% of the phenolic compounds present in beer are derived from malt, while the remaining 30–20% come from hops [5,6]. Moreover, malt can contribute to around 95% and 86% of the antioxidant capacity of dark and pale beers, respectively [7], while hopping did not affect significantly the antioxidant activity of beer [8].

The main antioxidant compounds in beer are phenolic compounds and melanoidins (formed throughout Maillard reaction (MR). In addition, some antioxidant additives used in beer (i.e., vitamin C) may also contribute to its antioxidant capacity [9,10].

The content of phenolic compounds and melanoidins in beer is greatly influenced by the genetic factor of its raw materials and therefore by the environmental conditions in which they grow and it is also influenced by technological brewing factors [11,12].

Beer production is an extremely complicated process since many variables are taking place and the chemistry and biochemistry involved in it are highly complex. Therefore, the compositions and concentrations of reducing substances are constantly changing throughout the process and raw materials and treatments applied are a source of variation as well. Slight changes in structure, or even conformational changes, can alter the antioxidant activity of a compound. But not only shelf-life is partly affected by the antioxidative status of the beer, it is known that antioxidant activity in beer plays a crucial role in providing flavor stability to beer [13].

**Figure 2:**
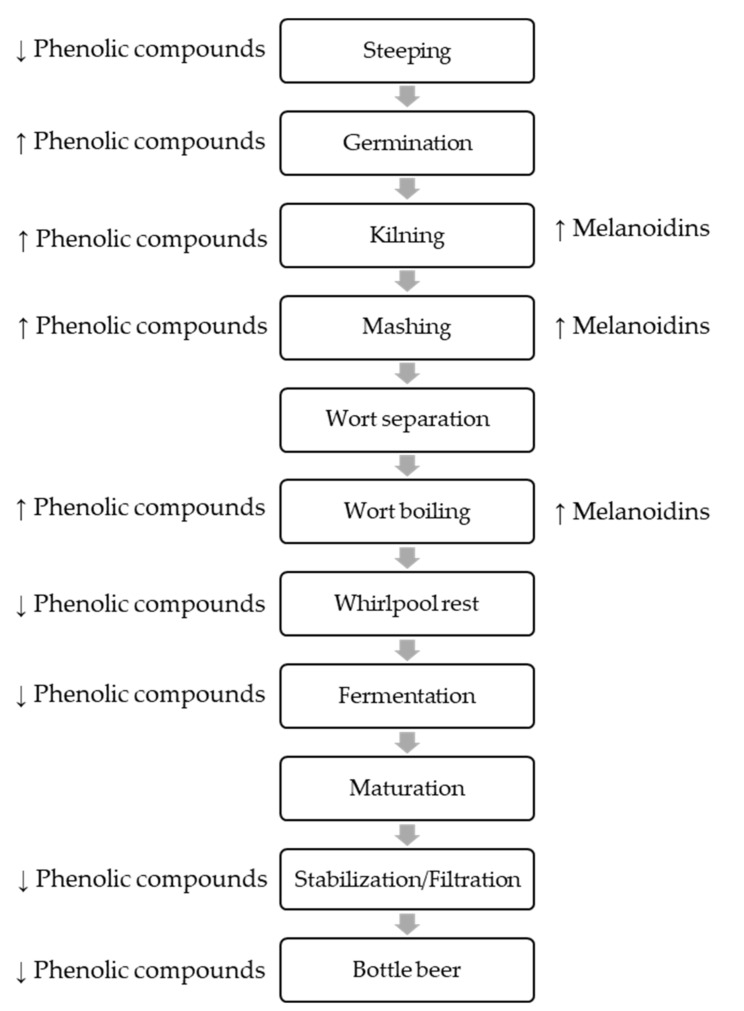
Schematic overview of the manufacturing process with special regard to changes in polyphenols and melanoidins content.

Thermal processing steps also induce important changes in the individual phenolic content of malt. For example, high temperatures can induce the degradation of phenolic compounds (including the phenolic acids) or their polymerization (as the proanthocyanidins). In fact, hot kilning regimens were shown to be responsible for a decrease of the levels of ferulic acid [14]. This can also be attributed to the formation of melanoidins during kilning. They are mainly present in dark malts and can trap polyphenols within its structure and decrease the content of these phenolic compounds [15]. Also, the thermal degradation of ferulic acid esterase and related enzymes, which are responsible for the release of bound phenolics from cell walls, may promote an overall decrease of phenolic acids with increasing the kilning temperature [14]. Figure 2 shows schematic overview of the manufacturing process.

## 2. Health Benefits of Beer and Antioxidants

### 2.1. Health Benefits of Beer Consumption

Moderate beer consumption has been shown to have beneficial effects on human health, many of which are based on the redox properties of the antioxidant compounds present in beer [5,16,17].

It is known that antioxidants present in beer help to improve certain diseases, for example, moderate beer consumption is associated with an increase in bone density, cardiovascular [18] and immunological benefits and is also associated with anti-inflammatory and antioxidant properties [19]. Moderate beer intake may also exert higher protection against coronary heart disease than spirits; it has been reported that systolic blood pressure, homocysteine, and several biomarkers of inflammation decreased only after the non-alcoholic beer intervention, and these effects are likely to be attributed to the non-alcoholic fraction of the beer, mainly polyphenols [20].

### 2.2. Health Benefits of Polyphenols

In regard to polyphenols, these are characterized by the presence in their structure of one or several phenolic groups, capable of reducing reactive oxygen species and various organic substrates and minerals. These redox properties explain the considerable interest in their role in the prevention of several major chronic diseases associated with oxidative stress, such as cardiovascular diseases, cancers, type II diabetes, neurodegenerative diseases or osteoporosis [21]. Phenols present in beer help lower blood pressure and increase the concentration of nitric oxide in the plasma, reducing the risk of cardiovascular disease [18].

These healthy properties are, in part, due to a specific type of phenolic compounds present in beer, the flavonoids. These compounds also possess anti-inflammatory, antioxidant and hypocholesterolemic properties [22]. In addition, polyphenols prevent oxidation of low-density lipoproteins [22] as they block free radicals that can oxidize fats in the body [23]. Polyphenols are recognized as preventers of colon cancer [24,25,26]. They are also able to cause positive changes in the gut microbiota, for example, flavonols induce an increase in the growth of *Lactobacillus* spp. and *Bifidobacterium* spp [24]. Polyphenols are also associated with improvements experienced by women in menopause [27] and improvements observed in people suffering from arthritis [28], but the bioavailability of polyphenol associated with this benefit, resveratrol, is low [29].

More specifically, some antioxidants have been studied such as xanthohumol (flavonoid present in beer and found only in hops [30]) and its cyclization product, isoxanthohumol, which have been studied previously by us [31] both present anti-cancer properties. Xanthohumol displays many bioactive effects such as antioxidant, anti-inflammatory, anti-microbial, hypoglycemic, and anti-obesity [32,33]. In particular, this compound is effective against different types of cancer [30] among which are: breast [34], ovarian [34], prostate [35], of colon [36] and pancreas [37] as well as being effective against leukemia [38] and protecting DNA against oxidative damage [39]. With a xanthohumol content of around 200 mg/L, beer is the principal source of this molecule in the human diet [33,40].

### 2.3. Health Benefits of Melanoidins

Regarding melanoidins, several works have shown that in addition to their ability to affect the color, flavor, and body of beer, these compounds can exert a certain effect on health. Difficulties in ascribing definite properties to individual melanoidins are caused by their diversity, complexity, drawbacks with purification and identification, and poor solubility in water and organic solvents. Additionally, other low molecular weight compounds are usually linked with melanoidins and may influence their properties. Furthermore, the degree of digestibility and bioavailability of melanoidins in organisms is often low [41].

Nonetheless, some studies have shown that melanoidins exert antioxidant, antimicrobial, antihypertensive, antiallergenic, and prebiotic properties [42]. Melanoidins also demonstrate the ability to bind metal ions such as Fe^+2^ [43] and are considered as antimutagenic and tumor growth-inhibiting compounds [44,45]. Melanoidins protect against damage caused by ROS to DNA and a more intense effect was found for dark beers than for blond beers due to dark beers are richer in melanoidins [41].

There is evidence that melanoidins behave as dietary fiber, being indigestible by humans and fermented in the gut, dietary melanoidins are not digested in the upper gastrointestinal tract and they are mainly recovered in the faeces [46]. Consequently, food melanoidins, as part of the food indigestible material that reaches the lower gut, can be metabolized by the gut microorganisms and have to be considered as a potential prebiotic material.

## 3. Phenolic Compounds in Beer

**Figure 3:**
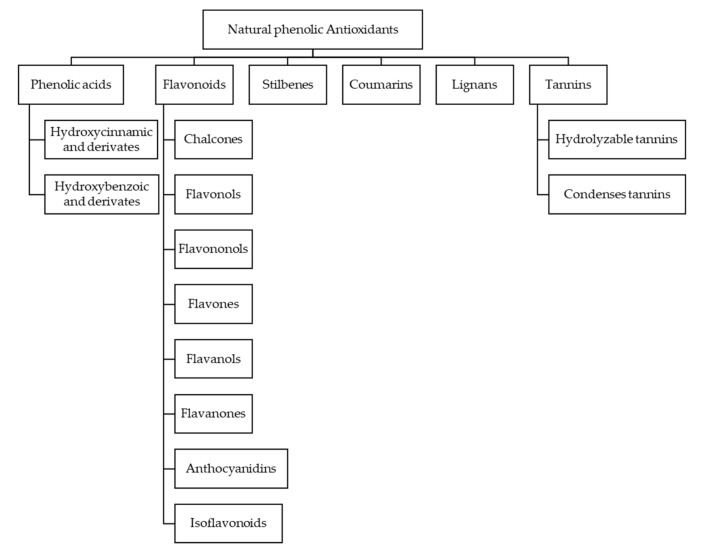
Natural phenolic antioxidants classification.

Phenolic compounds are a group of chemical substances characterized by the presence of at least one phenol unit. Studies have shown that the contribution to the antioxidant activity of beer from phenols is greater than 50% [47,48]. The classification of the different phenols is done according to their structure, so we have phenolics acids, flavonoids, stilbenes, coumarins, lignans, tannins, chalcones flavonols, flavononols, flavones, flavanols, flavanones, anthocyanidins and isoflavonoids [49,50]. An extended scheme can be seen in the Figure 3.

**Figure 4:**
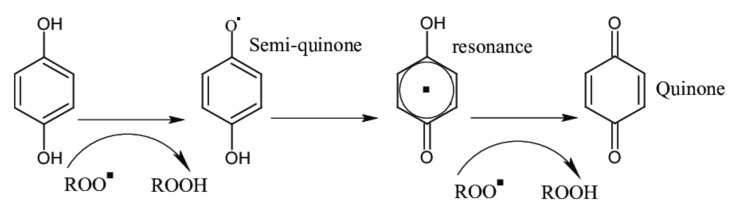
Basic scheme of radical scavenging by phenols.

The electronic configuration of the phenols allows easy release of electrons to free radical species, as seen in Figure 4. This release transfers the radical character to phenol, a radical that is generally more stable than the initial radical species [51].

When phenolic compounds react with free radicals, the reaction products are reduced radical species plus a phenolic radical. The antioxidant activity of phenolic acids depends on their chemical structure, especially on ring substituents, and it has been observed that homologous cinnamic acids exhibit a higher antioxidant potential than the respective benzoic acid derivatives [52]. The antioxidant activity of phenols depends on the number of OH substituents [53], also, recently Zhang et al. 2017 found that the antioxidant activity of proanthocyanidins depend on their degree of polymerization [54].

Phenolic compounds have their origin from barley (about 70–80%) and hop (about 30–20%) [55]. The differences between some beers and others are justified by the brewing steps [56,57], the genetic factors of the raw material used [18] and the malt and hops, the polyphenol content of which depends on the cultivation region, crop handling and processing [55,58].

Assays for phenolic compounds in beer can be classified as those determining the total phenolic content and those allowing the determination of the phenolic profile.

### 3.1. Total Phenolic Content Measurement

Total phenol content (TPC) is usually expressed as gallic acid equivalents (GAE) and there are several analytical methods for the quantification of the TPC in beers, most of them based on the reaction of the phenols with a colorimetric reagent and the measurement of the absorption of the colored product in the visible region of the spectrum [59]. These assays show very different specificity and the contribution of other groups of compounds must be often taken into account [59,60,61].

The most recognized classical methods for the determination of total phenol content are the Folin-Ciocalteu method (FC), Bishop’s method and the Prussian blue assay [59].

The Folin-Ciocalteu method is based on the reduction of the FC reagent, (mixture of phosphomolybdate and phosphotungstate) by the polyphenols and absorption measurement at 260 nm. The reduction reaction is enhanced under alkaline conditions [59,60,61]. Since color formation in the Folin-Ciocalteau reaction is based on the chemical reduction of the reagent.

A second method for the determination of the TPC is Bishop’s method, which is accepted as an official method by the European Brewery Convention (EBC) (EBC method 9.11). This is a spectrophotometric method based on a chelation reaction between the polyphenols and Fe (III) in basic medium, where the sample is mixed with carboxymethyl cellulose/ethylenediaminetetraacetic acid reagent (CMC/EDTA), and CMC is then added to improve transparency, whereas EDTA is used as an antiseptic. Finally, the absorbance at 600 nm is subsequently measured. When compared to FC assay, Bishop assay results tend to be more selective and, therefore, provide lower polyphenol concentrations.

Prussian blue assay is a good option to determine phenols because it has low interference by non-phenolic compounds. This method is based on the reaction of phenols with ferricyanide ion [Fe(CN)_6_]^3−^, the latter being reduced to ferrocyanide ion [Fe(CN)_6_]^4−^. Ferrocyanide ion reacts with an excess of iron (III) to form ferric ferrocyanide Fe_4_[Fe(CN)_6_]^3^, commonly known as Prussian blue, whose absorbance at 700 nm is measured [59].

Most modern methods to determine the TPC are based on the combined use of enzymes and electroanalytical techniques. Biosensors for polyphenol detection have been developed on the basis of enzymes such as tyrosinase, laccase and peroxidase, using different electrode materials, flow systems and sample pretreatment techniques with shorter time for analysis [62]. Phenols are enzymatically oxidized to quinones or radicals and then detected at the electrode by their reduction currents [63]. These methods have several advantages over traditional methods, such as simple instrumentation, sensitivity, quick response, no sample treatment, low cost, compact size, and the possibility of in situ and online analysis, attracting special interest from wineries. It has also been seen that there are no significant differences between the results obtained by these methods and the FC method [63,64,65].

It is known that total polyphenols and phenolic acids profile change among different beer types. In general, fruity and dark beers are the ones with the highest TPC values whereas alcohol-free beers have the lowest. Between these extremes are bock, abbey, ale and lager beers [55]. Several studies have shown that fruity beers present high phenolic content, since fruits are an important source of phenols. Values of 1033 mg GAE/L, measured by FC method, have been reached for a Belgian beer made with elderberries [66]. High values are also obtained for beers made with persimmons [67] or goji berries [68].

A second position in phenolic content corresponds to dark beers. Published studies show a minimum phenol content for this type of beer of 300 mg GAE/L reaching 943 mg GAE/L, a value obtained by Granato et al. 2011 for a beer aged with sour cherry [69]. Granato et al. 2011 also compared other dark beers in front of pale beers and the results obtained were similar to previous ones [55], obtaining higher values for dark beers (280–525 vs 119–200 mg GAE/L). Dark beer presents higher TPC due to the greater presence of malted barley and the generation of polyphenols during the malting process [30].

The lowest values for TPC are found in alcohol free beers [55,60]. Alcohol free beers are usually brewed with lower original wort extract and inhibition of alcohol formation, or as normal alcoholic beers, with alcohol removal at the last step [55]. The fact that alcohol-free beers present a lower TPC is important because it indicates that the beer was not developed with techniques that remove alcohol without altering the remaining compounds (phenols) [60], such techniques can be dealcoholisation of beer by reverse osmosis [70] or the use of membranes [71]. TPC for alcohol-free beer varies between 75 and 366 mg GAE/L [55,57,60,66,72,73].

Most studies are focused on the determination of the phenolic content in lager beers. In this sense, an extensive study was carried out by Zhao et al. in 2010 [47], in which 34 lager beers were analyzed and where TPC ranged between 152 and 339 mg GAE/L. Other later studies on Polish, Belgian, Serbian, Indian or Hungarian beers agree with that study [60,66,72,74,75]

In general, ale beers present higher TPC than lagers [76]. In a comparative study between different Brazilian beers, it was observed that brown ale beers presented higher TPC than lager beers and also higher antioxidant properties [69]. Similar results were obtained by Ditrych et al. [72].

### 3.2. Phenolic Compounds Profile

The phenolic acid profile of a beer depends on several factors such as the differences in raw materials, brewing process and original gravity [47,55]. 

Polyphenols can be used to identify beers in three different ways:

1. The qualitative phenolic indicator (its presence or absence can distinguish between different beers).
2. The quantitative phenolic indicator (some polyphenols are produced in beers of particular origin on a different level and therefore an upper and lower limit can be defined)
3. Proportions of phenolic compounds selected from each other: the so-called relative quantitative fingerprint [77].

As indicated above, the available methods for analyzing total phenols content are not specific for phenolic compounds since they can present interactions with other reducing substances, so the current trend is to analyze each compound individually for detection and quantification.

Due to high sensitivity and selectivity, high performance liquid chromatography (HPLC) is the technique most used for phenolic compound identification [52,59]. More advanced and complex techniques such as liquid chromatography coupled with an electrospray ionization hybrid linear ion trap quadrupole Orbitrap mass spectrometer (LC-ESI-LQT-Orbitrap-MS) have allowed quantifying up to 47 phenolic compounds [6].

The individual determination of the phenols present in beer is usually complex because these compounds are usually found in low concentration, therefore the concentration of the samples is a necessary and key stage. One possibility is the use of a previous stage of separation using cartridges followed by HPLC. With this methodology, phenolic acids derived from benzoic acids and cinnamic acid can be quantified, as well as flavonols [73]. In subsequent studies, this methodology was already applied to samples of conventional beers [47,55,60,78,79]. It is important to highlight that Manfroir et al. (2019) carried out simultaneous determination of nitrogen compounds and phenolic compounds using a single preparation stage. An alternative to the use of cartridges is to acidify the samples in order to perform a liquid-liquid extraction followed by HPLC [77,80].

More than 50 phenolic compounds have been identified in beer [75]. The most current collection of these compounds is the one published in 2018 [52]. The study of phenolic compounds in beer is continually providing phenolic compounds that had never been identified before, as is the case of Manfroi et al. 2019 work [79] in which 12 new phenolic compounds in beers are observed for the first time.

Most recent studies agree that the most abundant phenolic compounds in beer are ferulic [55], gallic [47] and p-cumaric acids [52], although vanillic and synaptic acids have also been described as important phenolic acids in this beverage [52]. Ferulic acid is the most abundant phenolic acid in European beers [55,73], Chinese [22,47] and Chilean [81], but in Brazilian or Serbian beers [60] gallic acid predominates [82].

According to the type of beers, in the ale type, it is the caffeic acid that is found in the highest proportion [58] and in lager-type beers it is gallic acid [47]. Ferulic acid predominates in non-alcoholic, black, abbey, wheat, pilsen and bock beers [55]. Xanthohumol is one of the most important antioxidants because of its health effects, reaching concentrations between 0.002 mg / L and 0.628 mg/L [22]. Roasted malts increase the content of this compound in beer [83]. Resveratrol, another polyphenol of great interest, is found to a lesser extent in lager beers or alcohol-free [79] than in ale-type beers [84].

## 4. Melanoidins

Melanoidins are macromolecular, nitrogenous and brown colored products of Maillard reactions, which are formed during the malting and brewing process [34,41].

**Figure 5:**
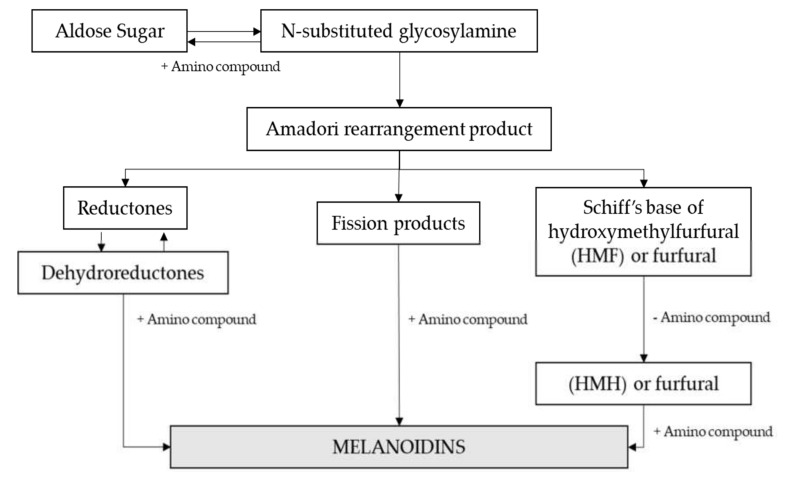
Maillard reaction scheme. Adapted from [85].

The Maillard Reaction (MR) is a complex network of reactions summarized in Figure 5. The initial stage involves the condensation of a carbonyl group, mostly form a reducing sugar, with a free amino group within peptides or proteins. This results in an Amadori rearrangement product, which can react further to give colored, low molecular mass products and melanoidins.

Maillard reaction products change depending on the time and temperature applied during the brewing process. The formation of high-molecular-weight (HMW) compounds occurs during the final stages of the Maillard reaction by polymerization of highly reactive intermediates [86,87]. Several works have demonstrated that malt roasting induces the polymerization of early-formed low-molecular-weight compounds (LMW) (< 10 kDa) into HMW brown compounds (> 300 kDa), reason why the content of LMW in roasted malts is lower than in pale malts [87,88]. Therefore, pale and caramel malts are characterized by light brown LMW colorants while roasted malts are characterized by intense brown HMW [88,89,90].

Two major factors limit the actual physiological relevance of the biological activities of melanoidins. First, the limited knowledge of the structure of food melanoidins makes it difficult to identify the active principles responsible for the specific biological activity. Secondly, although melanoidins are consumed regularly as part of the daily human diet, they are generally considered poorly absorbable and poorly bio-available compounds [89].

The structural properties of food melanoidins are largely unknown. However, it is assumed that it does not have a definite structure, which largely depends on various factors such as the nature and molar concentration of parent reacting compounds and reaction conditions as pH, temperature and heating time [91]. The prominent difficulty in the study of the structure of food melanoidins is a consequence of their diversity and heterogeneity [92].

### Measurements of Melanoidin Content

There are pitfalls in the quantitative analysis of melanoidins [10] and the usual way to account for melanoid content requires three steps: first an extraction is performed, followed by an ultracentrifugation to subsequently lyophilize the solution. The total amount of melanoidin is obtained from the yellow extract obtained [93,94].

According to published studies, the melanoidin content of the different beers would be: Dark>blond>alcohol free [93,94]. This content ranges from 0.58 mg/L for alcohol-free beer or 0.61 mg/L for blond beer to 1.49 for dark beers, these differences are attributed to the raw material and the particular brewing process [93,94].

Generally, black beer has a higher melanoidin content than blond beer, since black beers are brewed from a malt which is more toasted than in blond beers [3]. In general, pale malts provide less reducing power to the wort and beer than more colored malts. Kilning involves drying the malt for up to 30 h and results in a stable product that can be easily handled, stored and milled. The reducing power is associated with the higher temperatures involved in the production of color compounds, including melanoidin and phenolic species [95]. As a result of the higher temperatures applied to produce special dark malts, higher levels of antioxidants (reductones and melanoidins) are formed during MR. Consequently, beers with dark malts normally have a longer shelf life than pale beers [96].

**Figure 6:**
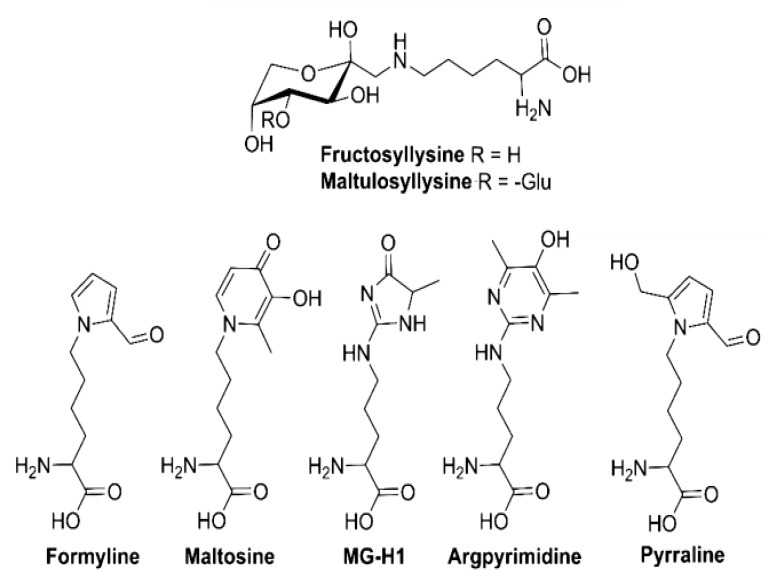
Structures of the several Maillard reaction products found in beer. Adapted from [97].

One interesting work on Maillard Reaction Products (MRPs) present in beer was carried out by Hellwing et al. 2016 [97]. They quantified seven MRPs in different types of beer (Pilsner, dark, bock, wheat, and nonalcoholic beers) by HPLC-ESI-MS/MS in the multiple reaction monitoring mode through application of the standard addition method (Figure 6). A high molecular weight fraction was isolated by dialysis and hydrolyzed enzymatically prior to analysis. Results concluded that the most important free MRPs in beer are fructosyllysine (6.8–27.0 mg/L) and maltulosyllysine (3.7–21.8 mg/L). In addition, the analyzed beers contained comparatively high amounts of late-stage free MRPs such as pyrraline (0.2–1.6 mg/L) and MG-H1 (0.3–2.5 mg/L) and minor amounts of formyline (4–230 μg/L), maltosine (6–56 μg/L), and argpyrimidine (0.1–4.1 μg/L) [97].

Other investigations carried out recently were able to isolate a low-molecular-weight yellow pigment from black beer. This pigment, identified as perlolyrine (which is a Maillard reaction product from tryptophan), was present in various kinds of beer at the level of 3.2–14.0 μg/100 mL [98].

There are various mechanisms by which Maillard reaction products may act as antioxidants: oxygen scavengers, reactive oxygen scavengers, reducing agents and metal chelating agents [92]. Traditionally, the use of colored malt is known to improve the stability of the finished beer, and it has also been shown that more-highly colored beers retain a greater reducing power during storage [87]. Several works carried out showed the existence of positive correlations between antioxidant activity and malt color, which have been ascribed to the presence of Maillard components [87,95]. More recently, Zhao et al. (2013) [94] also found positive correlations between melanoidin content in beers and antioxidant capacity.

## 5. Methods of Antioxidant Measurement

The reducing power is generally associated with antioxidant activity and could serve as a significant indicator of such activity. Some compounds show antioxidant activity through chelation of metal ions. Metal ions such as iron or copper can induce molecular oxygen to form reactive oxygen species that participate in the oxidation of beer and give rise to unpleasant flavors.

On the basis of reaction mechanisms involved in the free radicals reduction processes, the methods to determine the antioxidant capacity are divided into two general groups: Methods based on the Single Electron Transfer (SET) and methods based on the Hydrogen Atom Transfer (HAT) [99]. The result is the same: the inactivation of free radicals, however, the kinetics and secondary reactions involved in the process are different.

Single Electron transfer (SET): Y*XH* + *R*• ⟶ Y*XH*^+^• + *R*^−^

Hydrogen atom transfer (HAT): Y*XH* + *R*• ⟶ Y*X* • + *RH*

Y*XH*: Antioxidant, *R*•: Free radical

In SET methods, an electron is provided by the antioxidant to the free radical and then the antioxidant becomes a radical cation, whereas in HAT methods the free radical removes one hydrogen atom from the antioxidant, and the antioxidant becomes a radical [61]. 

Phenolic compounds can undergo both HAT and SET, way depends mainly on the chemical structure of the phenolic compounds. Tocopherol, followed by hydroxytyrosol, gallic acid, caffeic acid, and epicatechin are the compounds most likely to go by HAT. Resveratrol and kaempferol are better suited for SET [61]. Several works carried out have shown that gallic acid and caffeic acid are the main phenols present in beer [47], so it can be assumed that HAT is the antioxidant mechanism that occurs mostly in beer. Table 1 shows the methods of evaluation of the antioxidant activity in vitro.

The antioxidant activity is generally expressed as equivalents of trolox (TE) per liter (mmol TE/L). The DPPH, ABTS, ORAC, FRAP, TRAP methods are those that are routinely used for the measurement of antioxidant activity in beers due to their better sensitivity, convenience, and short assay times [10].

Several factors are involved in the antioxidant activity of different groups of compounds, the chemical structure of these compounds, the nature of the solvent, the temperature and pH, as well as the chemical structure of free radicals. Thus, to study antioxidant properties, at least three evaluation methods should be used: HAT, SET, and a combined method, HAT-SET. In addition, it is essential to perform reaction kinetics and to consider that in mixtures of antioxidant compounds, synergistic effects may be present, which could enhance the activity or even affect their reaction mechanisms [61].

FRAP values are strictly correlated with total phenolic content [55,101]. Using the FRAP method [10,94,102,103], it was shown that there are significant differences between the different types of beer. It is possible to establish a decreasing order in the antioxidant activity similar to the TPC which is the following:

dark> ale> lager> alcohol free

In general, dark beers have higher antioxidant activity [39]. The fact that dark beers have a high antioxidant activity may be due to the use of special malts such as caramel malts or malts with different colorations. During the boiling process of these malts, different Maillard compounds are generated that have antioxidant activity [66].

By regions, Belgian beers tend to have greater antioxidant activity than Portuguese beers [103]. Asian beers present a lower FRAP value than English or German beers, because generally Asian beers are less bitter [104]. Bitter beers have a higher antioxidant activity, bitterness comes from hops and this is responsible for certain phenolic compounds such as procyanidins, epicatechin or ferulic acid being released during beer brewing, increasing the antioxidant activity [104]. The main mechanism by which hop-derived acids act as antioxidants is by iron chelation and by scavenging radicals [105].

## 6. Perspectives and Concluding Remarks

In view of the benefits that antioxidants provide to beer and to human health, the new trends try to increase the content of antioxidants, developing beers with new characteristics.

One possibility to achieve this increase is to use rice malts for brewing and to get beers suitable for coeliacs. The total phenol content for this beer is 228 mg GAE/L, which is within the range of lager beers. The same goes for the values of FRAP, DPPH and ABTS studies for rice malt beers, which show an antioxidant activity that is comparable to lager barley malt beer [48].

Plants also can be incorporated, especially *A. heterophyllus*, *C. extensa, O. corymbosa* and *A. malaccensis*. These four plants are potentially rich sources of polyphenols and have good antioxidative properties [106]. Continuing with the plants, the use of *A. ruthenicum* results in lager beers with a TPC duplicated with respect to the same beer without adding this plant; the antioxidant activity of this beer was also improved with respect to the same beer without *A. ruthenicum*. The increase is caused because *A. ruthenicum* is an important source of proanthocyanins [107].

It is known that kefir has antioxidant properties, so the use of kefir during beer fermentation provides beers with a phenolic composition similar to beers made with *Saccharomyces cerevisiae*, kefir beer being the first produced by probiotics with as single fermenter [108].

Propolis is a natural product that has many functional properties such as antioxidant, antibacterial, anticancer, antifungal, anti-inflammatory and antiviral activities. The incorporation of propolis extract in beer reduces oxidation and fortifies the phenolic content that is typically reduced during different steps of the brewing process [109].

A new tendency is to use fruits or fruit juices, which present high content of antioxidants, so when added to beer, TPC and antioxidant activity increase. The use of carnelian cherry juice has doubled the TPC and antioxidant activity with the DPPH, ABTS and FRAP methods [110]. The incorporation of quince in beer increases its phenolic content [111]. Another possibility is the use of fruits such as persimmon [67], or goji berries [38,77]. In addition, the use of grapes has been reported to increase the TPC and antioxidant activity [112].

Based on the brewing process, a novel method consists in the use of hydrodynamic cavitation that allows to retain or generate a higher concentration of xanthohumol, desmethylxanthohumol and 6-geranylnaringenin [113]. It is also possible to use the remains of yeast used in the preparation as an ulterior source of phenols [114].

The use of new ingredients in the brewing process makes it possible to use the properties of these ingredients and to produce new beers whose antioxidant properties are increased compared to conventional ones. In addition, the sensory analysis data of these new beers are satisfactory [68,106], which indicates that there would be potential consumers for these new products.

## Figures and Tables

**Table 1:** Methods of evaluation of the antioxidant activity.

Method	Reaction Mechanism	Principle of Method	Type of Assay	Reference
Oxygen radical-absorbing capacity assay (ORAC) *	HAT	Antioxidant reaction with peroxyl radicals, induced by AAPH	Fluorescence	[10,61,100]
Lipid peroxidation inhibition capacity assay (LPIC) *	HAT	Antioxidant to inhibit peroxidation of lipids induced by a Fenton-like system	Colorimetry	[10,100]
Total radical trapping antioxidant parameter (TRAP) *	HAT	Antioxidant to scavenge luminol-derived radicals, generated from AAPH decomposition	Chemiluminescence quenching	[10,61,100]
Superoxide anion radical scavenging activity (SASA) *	HAT	Antioxidant to scavenge superoxide anion radical formation	Colorimetry	[10]
2,2′-azinobis (3-ethylbenzothiazoline-6-sulfonic acid) diammonium salt radical cation scavenging activity assay (ABTS) *	HAT	Antioxidant to scavenge an organic cation radical	Colorimetry	[10,61,100]
Hydroxyl radical averting capacity assay (HORAC) *	HAT	Antioxidant capacity to quench OH radicals generated by a Co (II) based Fenton-like system	Fluorescence	[10,100]
Cupric ion reducing antioxidant capacity assay (CUPRAC) *	SET	Cu (II) reduction to Cu (I) by antioxidants	Colorimetry	[10,100]
Ferric-reducing antioxidant power assay (FRAP) *	SET	Antioxidant reaction with a Fe (III) complex	Colorimetry	[10,61,100]
1,1-diphenyl-2-picrylhydrazyl radical scavenging activity assay (DPPH) *	SET	Antioxidant reaction with an organic radical	Colorimetry	[10,61,100]
Potassium ferricyanide reducing power method (PFRAP) *	SET	Potassium ferricyanide reduction by antioxidants and subsequent reaction of potassium ferrocyanide with Fe^3+^.	Colorimetry	[10,100]
Total oxyradical scavenging capacity total assay (TOSCA)	HAT	Ethylene concentration, generated during decomposition of α-keto-γ- methiolbutyric acid	Recording ethylene concentration relative to a control reaction	[61]
Ce (IV)-based reducing antioxidant capacity assay (CERAC)	SET	Reaction between Ce (IV) ion and antioxidants and subsequent determination of the produced Ce (III) ions	Fluorescence	[100]
N, N-dimethyl-p-phenylenediamine (DMPD)	Fenton type ET based reaction	DMPD radical cation (DMPD· +) is generated through a reaction between DMPD and potassium persulphate and is subsequently reduced in presence of antioxidants	Colorimetry	[100]
Crocin-bleaching assays (CBAs)	HAT	Abstraction of hydrogen atoms and/or addition of radical to the polyene structure of crocin.	Colorimetry	[61,100]
Thiobarbituric acid reactive substances (TBARS)	SET	Inhibition of production of TBARS from sodium benzoate under the influence of the free oxygen radicals derived from Fenton’s reaction	Colorimetry/Fluorescence	[100]
Metal-chelating activity assay (MCA) *	Other	Chelating ferrous ion with ferrozine	Colorimetry	[10]
Enhanced chemiluminescence (ECL) *	Other	Emission of light by substance that has absorbed light or other electromagnetic radiation of a different wavelength	Recording of fluorescence excitation/emission spectra	[10]
Electron spin resonance (ESL) *	Other	Free radicals by electron spin resonance	Recording the time of occurring of free radicals	[10]

* Antioxidant Activity Assays for Beers.
